# Ribosomal Proteins Control or Bypass p53 during Nucleolar Stress

**DOI:** 10.3390/ijms18010140

**Published:** 2017-01-12

**Authors:** Annapina Russo, Giulia Russo

**Affiliations:** Department of Pharmacy, University of Naples “Federico II”, 80131 Naples, Italy

**Keywords:** nucleolar stress, p53, uL3 (rpL3), apoptosis, ribosomal proteins, ribosome, nucleolus

## Abstract

The nucleolus is the site of ribosome biogenesis, a complex process that requires the coordinate activity of all three RNA polymerases and hundreds of non-ribosomal factors that participate in the maturation of ribosomal RNA (rRNA) and assembly of small and large subunits. Nevertheless, emerging studies have highlighted the fundamental role of the nucleolus in sensing a variety of cellular stress stimuli that target ribosome biogenesis. This condition is known as nucleolar stress and triggers several response pathways to maintain cell homeostasis, either p53-dependent or p53-independent. The mouse double minute (MDM2)-p53 stress signaling pathways are activated by multiple signals and are among the most important regulators of cellular homeostasis. In this review, we will focus on the role of ribosomal proteins in p53-dependent and p53-independent response to nucleolar stress considering novel identified regulators of these pathways. We describe, in particular, the role of ribosomal protein uL3 (rpL3) in p53-independent nucleolar stress signaling pathways.

## 1. Introduction

**Figure 1:**
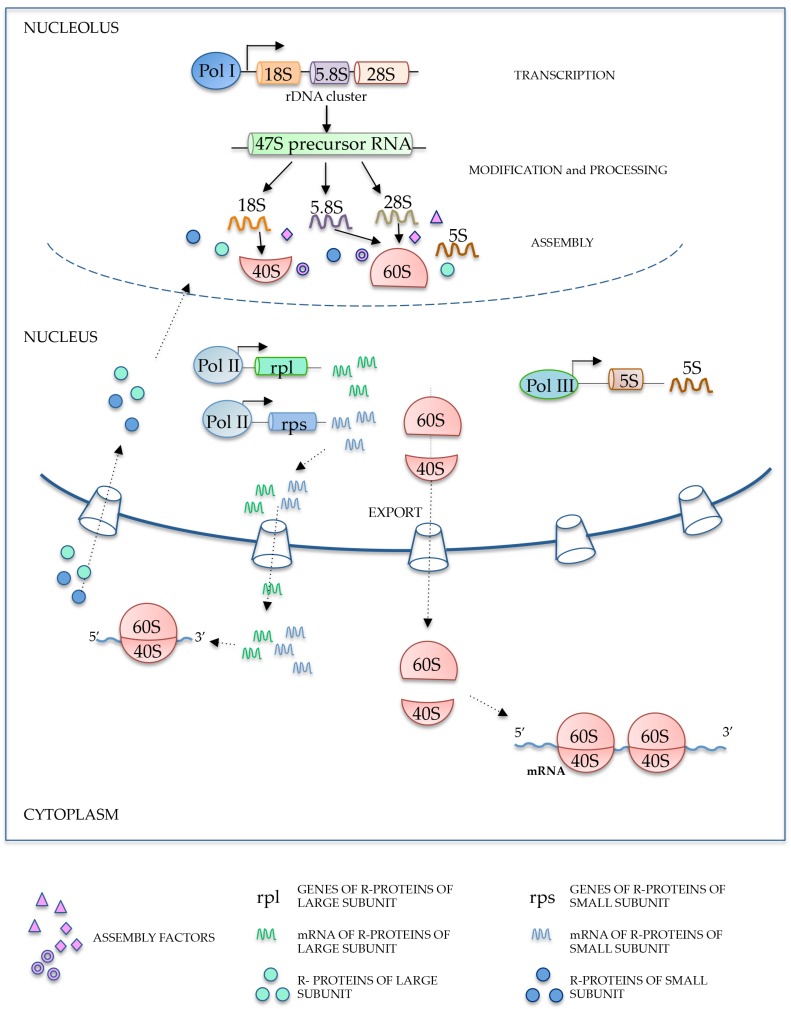
Schematic representation of ribosome biogenesis. rDNA: ribosomal DNA; Pol I: polymerase I; Pol II: polymerase II.

The nucleolus is a sub-nuclear compartment without membrane located within the cellular nucleus. In humans, it originates around the nucleolar organizer region (NOR) of five chromosomes (13, 14, 15, 21 and 22) each containing multiple repeats of ribosomal DNA (rDNA) transcription units [1,2]. The nucleolus is not present during all phases of the cell cycle; it is in fact present throughout interphase while it disassembles during mitosis. The nucleolus is the site of ribosome biogenesis, a complex process that requires the coordination of several events leading to nuclear export of the mature 40S and 60S subunits to the cytoplasm (Figure 1). These events include nucleolar transcription of rDNA by RNA polymerase I (Pol I) generating the 47S rRNA precursor (pre-rRNA), the nuclear import and nucleolar accumulation of ribosomal proteins (r-proteins), as well as nuclear transcription and nucleolar accumulation of 5S rRNA. The 47S pre-rRNA is subjected to specific modification to generate the mature 18S, 28S, and 5.8S rRNA (Figure 1). Thus, the biogenesis of eukaryotic ribosomes requires complex intracellular trafficking in order to ensure that all subunit components are present in the nucleoli so that efficient ribosome subunit assembly can occur [3]. Furthermore, this process requires the presence of hundreds of accessory or assembly factors represented by non-r-proteins and small nucleolar RNAs (snoRNAs) [3] and is generally coordinated with cell growth and sensitive to several cellular stimuli (Figure 1). Following a growth signal in the cell, an early event mediated by a target of rapamycin (TOR) signal-transduction pathway is represented by an increased ribosome production that allows cells to grow faster [4]. In this circumstance, newly synthesized r-proteins must rapidly be imported into the nucleus and accumulated at the nucleolar site for ribosome particle assembly, whereas free cytoplasmic r-proteins are degraded with a half-life of 2–3 min or less [5].

Import signals have been mapped for a number of r-proteins; a common feature is their very basic nature and greater complexity as compared with the classical nuclear localization signal (NLS) [6]. These proteins enter the nucleus by using non-classical pathways. Once in the nucleus, r-proteins are sequestered by multiple pathways that depend on the particular binding host within the nucleolus [7].

Ribosome biogenesis is the most demanding energetic and metabolic spending for the cells; consequently, this process is subject to severe quality control checks. When some defects in ribosome synthesis are revealed, some r-proteins through their extra-ribosomal functions [8] activate mechanisms of cell cycle arrest and/or apoptosis [9]. In humans, alterations of ribosome structure or function are involved in the development of cancer [10] as well as in several different diseases. For instance, mutations in r-protein (rp) S19 gene have been identified in about 25% of patients of Diamond-Blackfan anemia, a congenital hypoplastic anemia associated with various physical malformations [11].

Remarkably, deficiency of an r-protein does not lead to general deficits but to tissue-specific alterations leading to the hypothesis that ribosomes can regulate translation both quantitatively and qualitatively. It has been demonstrated that the affinity of ribosomes for a specific messenger RNA (mRNA) is unique. In condition of ribosome biogenesis defects, mRNA species with high affinity for translational apparatus will be translated preferentially with respect to those with low affinity. These events lead to consequent changes in gene expression [12,13,14]. Recent proteomic studies on highly purified preparations of human nucleoli have identified over 4500 proteins [5] as components of the nucleolar proteome, most of which are involved in activities different from ribosome biogenesis. In the last few years, it became evident that the nucleolus, by using these huge reservoirs of proteins, is able to regulate important cellular processes such as cell cycle progression, cellular proliferation and differentiation, DNA damage repair, genome organization, ageing, cell stress response, protein degradation, protein folding, and mRNA export [15]. These findings reinforce the idea that the nucleolus, beyond the production of ribosomes, plays a very important role in maintaining cell homeostasis by regulating events such as cell cycle progression and apoptosis [16,17]. It has been demonstrated that different events that disrupt ribosome biogenesis, i.e., UV and gamma radiation, oncogenes, nutrient and growth factor deprivation, rRNA or r-protein unbalance, hypoxia, and genotoxic agents, are associated with the activation of cellular processes regulated by the nucleolus, allowing the cells to adapt to the new environment [7]. This condition, defined as nucleolar stress or ribosome biogenesis stress, is able to activate nucleolar stress signaling pathways mediated by several r-proteins with consequent cell cycle arrest or apoptosis. Some of these pathways involve p53 tumor suppressor activity while others are p53-independent [16,17,18,19]. Of note, there is an emerging field of evidence suggesting the existence of a number of alternative nucleolar stress signaling pathways involving nucleolar proteins that are non-r-proteins [17]. It is also known that failure in the activation of these response pathways leads to the disruption of cell homeostasis contributing to the development of cancer or other pathologies [17]. The first indication that eukaryotic cells can trigger a previously unrecognized pathway in response to alterations in ribosome biogenesis comes from a study reporting that deletion of the gene encoding for eS6 (rpS6) in the liver of adult mice led to a block of 40S ribosome subunit biogenesis and inhibition of cell proliferation after partial hepatectomy [20]. Following this, a number of studies have demonstrated that defects in ribosome biogenesis upon inhibition of transcription or processing of pre-rRNA [21] decreased expression of proteins involved in ribosome biogenesis including specific r-proteins [22,23,24] thus activating the p53-mediated response to nucleolar stress.

Recently, some evidence has indicated that different drugs widely used for the therapy of a variety of solid tumors, including 5-fluorouracil (5-FU) [25], actinomycin D [26], oxaliplatin [27] and niclosamide [28], are able to induce nucleolar stress with the consequent release from the nucleolus to the nucleus of some r-proteins that, exerting extra-ribosomal functions, are able to activate p53-dependent or -independent nucleolar stress signaling pathways.

## 2. Role of Ribosomal Proteins in p53-Dependent Response Pathways to Nucleolar Stress

In eukaryotes, r-protein expression is regulated by multiple control mechanisms mostly at post-transcriptional and translational levels in order to maintain ribosome biosynthesis at the level appropriate to the requirements and growth conditions of the cell [29,30]. However, several reports indicate that r-proteins, in addition to their role as components of translation machinery, exert a variety of extra-ribosomal functions for which additional and specific regulatory strategies are required [29,30,31].

Alteration in the expression of r-proteins seems to correlate with tumorigenesis since many r-proteins are overexpressed in solid tumors and leukemia cells [32,33]. Tumor modeling studies in mice have demonstrated that tumors overexpressing uS5 (rpS2) can be eradicated by therapeutically targeting this gene [34]. On the other hand, quantitative reverse transcription polymerase chain reaction (RT-PCR) analysis of mRNA levels in some tumors versus mRNA levels in normal tissues showed a reduction of r-protein mRNA amounts [9,17]. These data suggest that some r-proteins might have a role as tumor suppressors, as described in zebrafish [35]. In addition, modifications in the expression of some r-proteins seem to modulate cellular drug sensitivity. In gastric cancer cell lines, uS15 (rpS13), uL14 (rpL23) and eL6 (rpL6) are able to suppress drug-induced apoptosis and promote drug resistance [36], whereas eS1 (rpS3a) synergizes with drugs to induce apoptosis [36].

**Figure 2:**
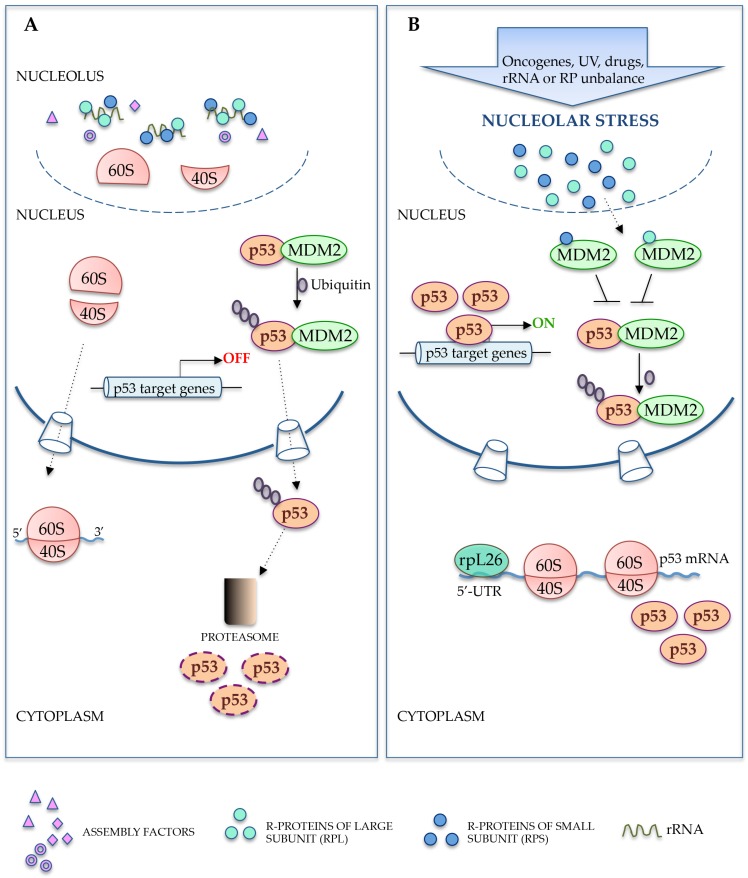
Role of ribosomal proteins in p53 activation upon nucleolar stress. (**A**) Under normal growth conditions, r-proteins are assembled with processed rRNA into 40S and 60S subunits in the nucleolus. p53 activity is maintained at low levels by its degradation promoted by mouse double minute (MDM2)-mediated ubiquitinylation; (**B**) During nucleolar stress, r-proteins are released into the nucleoplasm where they interact with MDM2 inhibiting its ubiquitin ligase activity and promoting the accumulation of p53. In the cytoplasm, ribosome-free rpL26 binds to 5′-untranslated region (UTR) of p53 mRNA to induce p53 translation. Accumulated p53 activates the expression of its target genes involved in the activation of events leading to cell cycle arrest and apoptosis.

Recent studies have shed new light on the mechanisms underlying the regulation of multiple coordinated events leading to ribosome biogenesis and revealed new connections between ribosome biogenesis and cell cycle [36]. The signaling pathways able to connect these two processes have long been relatively unknown; however, studies over the last decade have widely demonstrated the role of r-protein-MDM2 (mouse double minute)-p53 stress response pathway in the regulation of these events [17,18,19]. In response to events inducing nucleolar stress, several r-proteins from the large and small subunits translocate to the nucleoplasm and bind to MDM2, thus promoting p53 stabilization and subsequent p53-mediated cell cycle arrest or apoptosis [37,38,39] (Figure 2). Fumagalli et al. [24] report a more detailed analysis of the role of a subset of these r-proteins i.e., eS7 (rpS7), uL18 (rpL5), uL5 (rpL11) and uL14 (rpL23) on p53 response. They suggest that neither uL18 (rpL5) nor uL5 (rpL11) alone are able to suppress MDM2, but these two proteins work in a complex to regulate MDM2 activity. Furthermore, they demonstrate that the upregulation of p53 in response to depletion of eS7 (rpS7) or uL14 (rpL23) is mediated by uL5 (rpL11) and uL18 (rpL5) since depletion of either uL5 (rpL11) or uL18 (rpL5) completely abolish this response. Furthermore, depletion of other r-proteins does not suppress the p53 response elicited by depletion of eS7 (rpS7) or uL14 (rpL23), thereby indicating that this effect is specific for uL5 (rpL11) and uL18 (rpL5). These results clearly indicate that uL5 (rpL11) and uL18 (rpL5) are essential for p53 upregulation in response to impaired ribosome biogenesis.

According to these findings, a recent paper [40] reports the effect of depletion of each of the 80 human r-proteins on pre-RNA processing, nucleolar structure and p53 levels. The authors reported that most r-proteins (50/80) have no role either in nucleolar structure maintenance or control of p53 levels, while depletion of 21 r-proteins has no effect on nucleolar structure but gives rise to an increase in p53 levels. Furthermore, they found that eight r-proteins have a role either in p53 homeostasis or in nucleolar structure maintenance. In this scenario, uL5 (rpLl1) and uL18 (rpL5) have a unique behaviour, since their depletion greatly alters the nucleolar structure without inducing p53. Of note, depletion of any one of the r-proteins able to regulate p53 levels together with depletion of either uL5 (rpL11) or uL18 (rpL5) has no effect on p53 homeostasis indicating that the activity of r-proteins on p53 is dependent on the presence of these two proteins. Results reported in this paper confirmed a previous observation [24] that the disruption of the nucleolus is not a prerequisite for the activation of a nucleolar stress pathway associated with p53 upregulation. In fact, the authors found that a group of r-proteins are able to mediate the stabilization of p53 without nucleolar disruption.

The central role of uL18 (rpL5) and uL5 (rpL11) in a p53-dependent nucleolar stress signaling pathway has also been reported in a paper from Sloan et al. [41]. The authors demonstrated that uL18 (rpL5) and uL5 (rpL11) regulate p53 levels together with 5S as part of a ribosomal subcomplex, the 5S ribonucleoprotein particle (5S RNP). They found that when ribosome biogenesis is inhibited, a free trimeric 5S RNP accumulates in the nucleoplasm, before binding to MDM2 with consequent upregulation of p53 levels. In untreated cells, the 5S/uL18 (rpL5) complex is present in the nucleoplasm while uL5 (rpL11) is present exclusively in the cytoplasm and nucleolus; after the block of ribosome biogenesis induced by actinomycin D treatment, uL5 (rpL11) translocates into the nucleoplasm and associates with 5S/uL18 (rpL5) complex to form 5S RNP thus regulating p53 levels. The authors reported that 5S SRNP were also involved in cellular response to oncogenic stress induced by overexpression of tumor suppressor p14^ARF^ demonstrating a central role of this trimeric complex in many cellular stress signaling pathways. Nishimura et al. [42] demonstrated a new role of 5S RNP in inducing a p53-mediated cellular senescence in response to oncogenic and replicative stress. In addition to the above-described p53-dependent nucleolar stress signaling pathways, recent findings highlight the intricate interplay between p53, MDM2, and some r-proteins demonstrating the complexity of the reciprocal regulation between these key regulators of cell growth and cell death. The e7S (rpS7) functions as both regulator and substrate of MDM2 and regulates apoptosis [39]; uS3 (rpS3) binds to p53 or MDM2 [43]. Other r-proteins are able to directly stimulate p53 translation independently from MDM2. uL24 (rpL26) binds to p53 mRNA and enhances p53 translation in response to DNA damage. The regulatory role of uL24 (rpL26) on the p53 expression level is controlled in turn by MDM2 [44]. On the contrary, mitochondrial rpL41 does not interact with MDM2 or p53, but is able to induce p53-dependent apoptosis by triggering p53 translocation to the mitochondria [45]. Furthermore, some r-proteins directly involved in feedback regulation of the p53-MDM2 axis are also regulated by p53 at the transcriptional level. To date, eS25 (rpS25), eS27 (rpS27) and eS27L (rpS27L) were identified to be transcriptional targets of p53. In fact, p53 directly interacts with eS25 (rpS25), eS27 (rpS27) and eS27L (rpS27L) promoters and negatively affects their activity [46,47]. Moreover, degradation of eS27L (rpS27L) requires MDM2. In particular, eS27L (rpS27L) competes with p53 for binding the RING or acidic domain of MDM2. Similar to uL24 (rpL26) [44] and eS7 (rpS7) [39], eS27L (rpS27L) directly binds to MDM2 and serves as the substrate of MDM2 [47].

## 3. Role of Ribosomal Proteins in p53-Independent Response Pathways to Nucleolar Stress

It is known that most cancer cells contain mutant p53 or no p53 at all [48,49]. In recent years, some emerging evidence has indicated that p53 is not the only factor controlling the relationship between ribosome biogenesis and cell proliferation, thus revealing the existence of additional mechanisms that are p53-independent and involve r-proteins and nucleolar proteins, such as p14^ARF^ and NPM (nucleophosmin) that link nucleolar stress to cell cycle arrest and apoptosis [50].

### 3.1. uL18 (rpL5), uL5 (rpL11), uS11 (rpS14) and c-Myc

**Figure 3:**
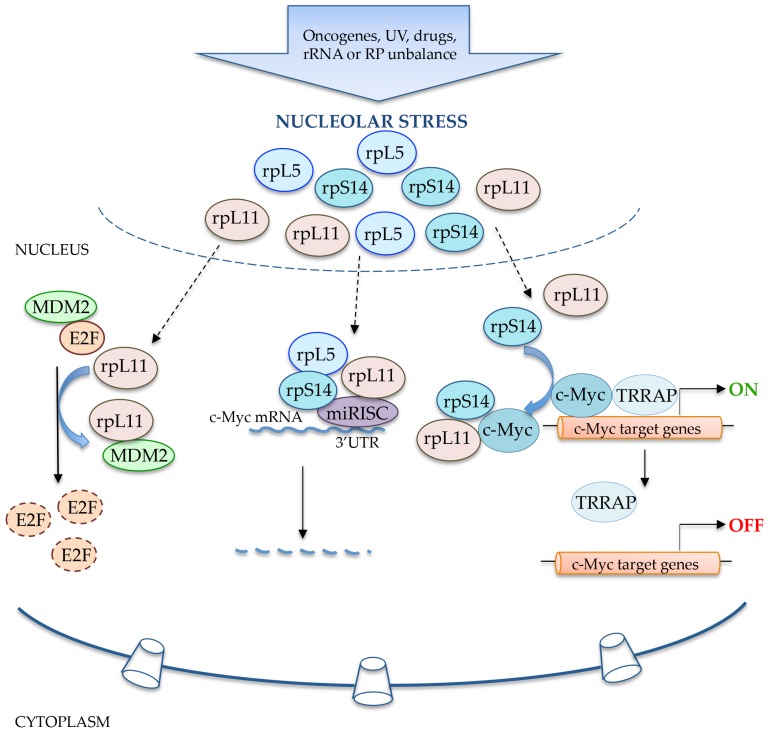
p53-independent and r-protein-dependent response pathways to nucleolar stress.

The oncoprotein c-Myc is a crucial transcription factor which controls the expression of numerous genes involved in cell growth and proliferation [51]. It has been reported that c-Myc activation is required for essential processes in the nucleolus. In fact, c-Myc transcriptional targets are involved in crucial steps of the ribosome biogenesis including the synthesis of rRNA, r-proteins and some translation initiation factors such as eukaryotic initiation factor 4E (eIF4E). Recent reports indicate that some r-proteins negatively affect c-Myc functions by directly repressing its transcriptional activity and/or inducing its degradation through the miRNA-mediated molecular mechanism [52]. During ribosomal biogenesis, uL18 (rpL5) and uL5 (rpL11) are two critical feedback modulators of c-Myc expression [53]. These two proteins form a complex with c-Myc mRNA and act in a cooperative manner to repress c-Myc expression, thus preventing the recruitment of c-Myc and its cofactor TRRAP (transformation/transcription domain-associated protein) to the promoters of c-Myc target genes such as nucleolin and E2F2 (E2F transcription factor 2, Figure 3). uL5 (rpL11) directly binds to c-Myc MBII domain [53] but also recruits of micro-RNA-induced silencing complex (miRISC) with miR-24 or miR-130a to c-Myc mRNA at its 3′ untranslated region (3′-UTR), leading to c-Myc mRNA degradation [54]. uL18 (rpL5) cooperates with uL5 (rpL11) in suppressing the expression of c-Myc through a RISC-mediated miRNA targeting pathway (Figure 3). In particular, uL18 (rpL5) requires two RISC components for its inhibitory function, TRBP (HIV-1 TAR RNA-binding protein) and Ago2 (Argonaute 2). Furthermore, it has been recently demonstrated that in esophageal squamous carcinoma cells, the depletion of 5S rRNA by miR-150 and miR-383 enhances the interaction between uL5 (rpL11) and c-Myc leading to the inhibition of Myc activity [55]. In addition to uL18 (rpL5) and uL5 (rpL11), uS11 (rpS14) also functions as a negative regulator of c-Myc [56]. Similar to uL11 (rpL5), uS11 (rpS14) is able to bind to the MBII domain of c-Myc, impairing the recruitment of the cofactor TRRAP to its target promoters, and induces c-Myc mRNA degradation through the interaction with miR-145 and Ago2-mediated pathway, thus resulting in the inhibition of c-Myc-induced cell proliferation (Figure 3). In addition, uS11 (rpS14), differently from uL5 (rpL11), associates with the bHLH-LZ domain, which is important for the binding of c-Myc to DNA and Max (MYC-Associated Factor X) and negatively influences the c-Myc-Max interaction [56].

Upon nucleolar stress, ribosome-free rpL11 interacts with MDM2 thus promoting E2F-1 degradation; ribosome-free rpS14, rpL5 and rpL11 bind to 3′-UTR of c-Myc mRNA to enhance the recruitment of miRNA-induced silencing complex (miRISC) for c-Myc degradation; rpS14 and rpL11 negatively regulate c-Myc transcription activity. They bind to c-Myc box II domain and inhibit the recruitment of c-Myc co-activator TRRAP to c-Myc target gene promoters thus suppressing cell proliferation.

### 3.2. uL5 (rpL11) and E2F-1

E2F-1 (E2F transcription factor 1) is a transcription factor belonging to a family of transcriptional regulators called the E2Fs, which controls the expression of genes whose products are important for the entry and passage throughout the S phase [57]. E2F-1 is a key mediator of p53-dependent [58] and p53-independent apoptosis. In the p53-independent pathway, E2F-1, once activated, positively regulates some crucial pro-apoptotic genes including *p73* [59], *Apaf-1*, *PUMA*, *Noxa*, *Hrk/DP5*, and *Bim* [60]. Among the regulators involved in p53-independent activation of E2F-1, also the r-protein uL5 (rpL11) has been identified. It has been demonstrated that MDM2 prolongs the half-life of the E2F-1 protein, independently of p53 by preventing its degradation via ubiquitin [61]. In cells in which p53 is inactive, upon inhibition of rRNA synthesis, free uL5 (rpL11), released from the ribosome, binds to MDM2 causing the release of E2F-1 and its subsequent degradation (Figure 3). Downregulation of E2F-1 expression is associated with the inhibition of cell proliferation [57].

## 4. Other p53-Independent Response Pathways to Nucleolar Stress

There is an emerging field of evidence suggesting the existence in the cells of a number of alternative nucleolar stress pathways involving nucleolar factors that bypass p53 and directly play crucial roles in apoptosis. These p53-independent regulators of apoptosis include several factors, including nucleolar phosphoprotein B23 (NPM), Wnt target Peter Pan (PPAN) and ARF [19].

### 4.1. Nucleolar Phosphoprotein B23-Bc12-Associated X Protein (BAX)

NPM/Nucleolar phosphoprotein B23 is a nucleolus-associated protein able to interact and modulate MDM2 functions [62,63]. NPM is involved in the control of cell cycle progression, centrosome duplication, and genomic instability. Alteration of NPM functions through mutations or translocations may contribute to oncogenesis [64]. NPM interacts directly with the tumor suppressor p53 increasing its stability and transcriptional activation [62]. In addition, the cellular activities of NPM are tightly regulated by multiple factors that seem to be specific for each function; post-translational modifications, oligomerization and hetero-oligomerization strongly influence the cellular functions of NPM. In particular, emerging evidence indicates a functional correlation between NPM and some r-proteins, independent from ribosome biogenesis or its assembly [65]. To date, uL23 (rpL23) binds and sequesters NPM, a co-activator of Myc-associated zinc-finger protein (Miz-1), into the nucleolus thus controlling cell proliferation [66].

Recent studies describe a novel death evasion pathway that requires Bcl2-associated X protein (NPM-Bax) and is independent of p53 function [67]. In conditions of nucleolar stress, NPM is induced and relocalizes in the cells translocating from the nucleolus to the cytoplasm where it complexes with Bax, a crucial effector of mitochondrial apoptosis. Cytosolic NPM inhibits the activation and the translocation of Bax to mitochondria. NPM-Bax interaction effects associate with cell resistance to death stimuli [67].

### 4.2. Wnt Target Peter Pan-NPM-BAX

Recently, a novel p53-independent response pathway to nucleolar stress has been identified and involves PPAN [68]. PPAN belongs to the conserved Brix-domain protein family and contains different domains targeted to different intracellular compartments: nucleolus, cytoplasm, and mitochondria. In the nucleolus, PPAN, together with its interaction partner PES (Pescadillo), controls the maturation of the large ribosomal subunit in the nucleolus [69].

Following drug-induced nucleolar stress, PPAN translocates from the nucleolus and accumulates in the cytoplasm. This is accompanied by phosphorylation and subsequent cleavage of PPAN by caspases. The loss of PPAN induces NPM and upstream-binding factor (UBF) degradation and BAX stabilization and activation, which is followed by mitochondrial depolarization. These data indicate that PPAN is required to inhibit the mitochondrial apoptosis acting as a pro-survival factor and the authors assumed that PPAN may act upstream of the NPM-BAX pathway [69].

Altered ribosome biogenesis caused by PPAN knockdown results in p53 accumulation that initiates p53-mediated apoptosis program [70]. Of note, in PPAN-silenced cells, p53 depletion does not alter poly (ADP-ribose) polymerase (PARP) cleavage nor the mitochondrial depolarization, indicating that PPAN-mediated apoptosis functions independently of p53.

Nucleolar stress induction by chemotherapeutic agents is a promising approach for cancer treatment. Moreover, the silencing of PPAN sensitizes cells to the chemotherapeutic drugs such as actinomycin D indicating that PPAN is involved in cell response to drug-induced nucleolar stress response [69]. The authors assumed that in cells treated with actinomycin D, PPAN is exported for degradation to robustly trigger the apoptotic response. Nevertheless, the possibility that the observed increased efficacy of drugs was due to the alteration of ribosome biogenesis induced by PPAN deletion cannot be excluded.

These data identify PPAN as a possible target for the treatment of NPM-overexpressing cancers lacking functional p53 [68].

### 4.3. p14^ARF^

The human *Ink4a*/*Arf* (*Cdkn2a*) locus encodes for both the cyclin-dependent kinase inhibitor p16^INK4A^ and the p14^ARF^ tumor suppressor [71]. p14^ARF^ localizes into the nucleolus and interacts with NPM [72]. It is involved in maintaining nucleolar structure and limiting protein synthesis. In fact, the loss of Arf results in an increase in both number and size of NORs in mouse embryonic fibroblasts. Arf is a tumor suppressor which acts as key activator of the p53 stress signaling pathway. Oncogenes, such as *Ras* and *E2F*, induce the expression of Arf leading to p53-dependent cellular response. In fact, Arf is an upstream regulator of p53. It relocalizes MDM2 to nucleoli and prevents MDM2-mediated ubiquitination and degradation of p53 leading to its activation [72]. Although the Arf/p53 pathway has been shown to modulate many biological functions, recent findings show that Arf may act independently of the MDM2-p53 axis in tumor surveillance. The enforced expression of Arf induces cell cycle arrest and/or triggers apoptosis in cells lacking p53. Several studies reported that Arf binds and antagonizes the transcriptional function of *c-Myc* and *E2F1* oncogenes required for cell cycle progression in absence of p53 [73]. In addition, both *c-Myc* and *E2F-1* induce Arf expression. This is an example of p53-independent negative feedback mechanism. Arf negatively controls the cell growth independently of p53 also by activating ATM/ATR/CHK signaling pathways following exposure to genotoxic drugs [73].

## 5. p53-Independent-Mediated Response Pathways to Nucleolar Stress

### 5.1. Role of uL3 (rpL3) in the Ribosome

uL3 (reported in the text and in the figures as rpL3) is a component of the large subunit of cytoplasmic ribosomes evolutionarily conserved. Analysis of rpL3 in yeast ribosome synthesis indicates that rpL3 plays a crucial role in the formation of early pre-60S r-particles [74]. rpL3 contains two tightly packed globular domains bound on the solvent side of the large subunit, close to the binding region for GTP-dependent translation factors. In addition, rpL3 contains two extensions that enter deep into the central core of the large subunit and one of these is very close to the peptidyl transferase center (PTC) [75]. Meskauskas and Dinman have described rpL3 as the “gatekeeper to the A-site” [76]. In addition, a recent study shows an important role of rpL3 in the function of eIF5B in stimulating 3′ end processing of 18S rRNA in the context of 80S ribosomes that have not yet engaged in translation [77].

### 5.2. Post-Transcriptional Regulation of rpL3 Expression

rpL3 belongs to a subset of r-proteins that function not only within the ribosome participating in translation but also as extra-ribosomal players involved in a number of cellular events [26,78,79]. The expression levels of these r-proteins require additional and specific regulatory strategies and among these the autoregulation represents an efficient mechanism to control the level of a single protein. rpL3 autoregulates its own expression through the association of alternative splicing and nonsense-mediated mRNA decay (AS-NMD) [8]. We identified an alternatively spliced nonsense codon-bearing transcript from the human *rpL3* gene that is a natural target of NMD. This isoform originates from alternative splicing that causes partial removal of intron 3 in *rpL3* gene. The resulting mRNA isoform carries an intronic sequence that contains a PTC and is stabilized by NMD inhibition. In fact, this transcript is degraded by NMD [8].

The quantity of ribosome-free rpL3 acts as a sensor of its own expression either by downregulating canonical splicing or by upregulating non-canonical splicing. Under normal growth conditions, the canonical transcript is preferentially spliced; following an excess of rpL3, the canonical splicing is inhibited and the splicing is mainly directed towards the alternative mode. The result of this event is an increase in the level of alternative mRNA, which is targeted by NMD, and a consequent reduction in the level of rpL3 [8].

This negative feedback controls the levels of ribosome-free rpL3 and avoids wasteful production of the protein. The autoregulatory circuit of human rpL3 expression requires heterogeneous nuclear ribonucleoprotein H1 (hnRNP H1), K-homology splicing regulatory protein (KHSRP) and NPM [78,79]. hnRNP H1 binds to the *cis*-acting regulatory elements “G runs” in rpL3 intron 3, and through the cooperation with other splicing factors, it promotes the selection of the alternative splice site [78,79]. Unlike hnRNP H1 and KHSRP, NPM behaves as a negative regulatory factor of *rpL3* gene alternative splicing [79].

### 5.3. p53-Independent and rpL3-Dependent Response Pathways to Nucleolar Stress: Effects on Cell Cycle, DNA Repair and Mitochondrial Apoptosis

The action of ribosome-free rpL3 has been intensively studied by our group and the results highlighted its favourable anticancer potential over different conventionally used chemotherapeutic drugs such as actinomycin D, 5-FU, oxaliplatin and niclosamide [26,28,37,80]. The key extra-ribosomal role of rpL3 is to arrest cell cycle progression and/or to induce apoptosis in response to drug-induced nucleolar stress. rpL3 can induce G1 arrest through the activation of the cyclin-dependent kinase inhibitor p21 gene transcription and apoptosis by molecular pathways involving p21 [26,37,81]. In addition, upon nucleolar stress, rpL3 becomes a regulator of DNA repair in p21-dependent and p21-independent manner. In particular, our results show that rpL3 has a strong effect in inhibiting the precise non-homologous end joining (NHEJ) known as classical end joining [37].

**Figure 4:**
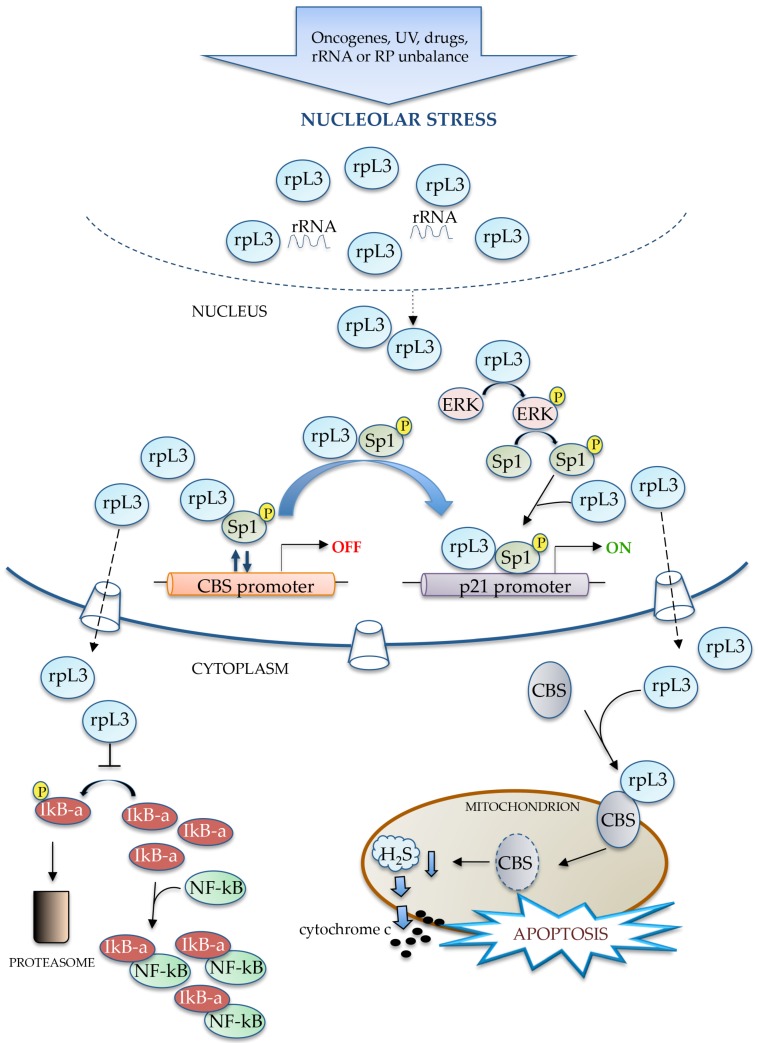
Model of p53-independent and rpL3-dependent response pathways to nucleolar stress. CBS: cystathionine-*β*-synthase.

During nucleolar stress, induced rpL3 accumulates in a ribosome-free form in the nucleus where it works as a transcription factor with dual opposite effects on *p21* and *CBS* (cystathionine-*β*-synthase) gene expression [80] (Figure 4). Ribosome-free rpL3 is able to induce the phosphorylation of extracellular signal-regulated kinase (ERK) which in turn activates downstream protein targets [82]. We speculate that the MEKs/ERKs pathway activated by free rpL3 could induce, in turn, the phosphorylation of Sp1 and could promote Sp1/rpL3 binding to the *p21* promoter [26]. Furthermore, rpL3 is able to bind to phosphorylated Sp1 and we have hypothesized that this interaction is essential to displace Sp1 from the *CBS* promoter thus inhibiting *CBS* transcription (Figure 4). At the same time, rpL3 recruits phoshorylated Sp1 on the *p21* promoter leading to the activation of *p21* expression. In the cytoplasm, ribosome-free rpL3 acts as a regulator of the stability of the protein product of its target genes *p21*, *CBS* and also of *IκB-α* [80] (Figure 4). Indeed, rpL3 prolongs the half-life of p21 protein by controlling the interaction between p21 and its negative regulator MDM2 [26]. On the contrary, rpL3 negatively affects CBS, one of three principal enzymes involved in the biosynthesis of H_2_S [83,84] using cysteine as substrate in various mammalian cells and tissues [85]. Analysis of the functional role played by rpL3 in the changes of CBS amounts revealed that rpL3 associates with CBS to shuttle it into the mitochondria for degradation (Figure 4). This effect is associated with decreased catalytic activity of CBS. The consequent decrease in H_2_S levels correlates with a decrease of the Bcl-2/Bax ratio, cytochrome c release from mitochondria and caspase activation leading to apoptosis [80]. Furthermore, ribosome-free rpL3 has also been identified as a p53-independent regulator of nuclear factor kappa-light-chain-enhancer of activated B cells (NFκB), a key factor with a role as a pivotal link between inflammation and cancer [86,87]. rpL3 inhibits NFκB activation by preventing NFκB nuclear translocation through the stabilization of the IκB-α protein, which is an inhibitor of the canonical NFκB pathway (Figure 4).

All these findings have extended the scenario of mechanisms of drugs such as actinomycin D, 5-FU, and oxaliplatin that specifically impact ribosome biogenesis for the treatment of cancers lacking active p53, thereby highlighting the importance of human rpL3 as a critical mediator of cell response to chemotherapy.

## 6. Remarks and Perspectives

Emerging data are accumulating regarding the identification of therapeutic targets [88,89,90] including those involved in ribosome biogenesis machinery such as r-proteins. To date, uS5 (rpS2) was reported to be a therapeutic target for the eradication of prostate cancer in preclinical tumor modeling studies [34]. Depletion of eL19 (rpL19) suppressed the aggressive phenotype of human prostate cancer [91]. rpL3 acts as a stress-sensing molecule essential in the cell response to drug-induced stress in cancer cells lacking active p53. In fact, the loss of rpL3 makes chemotherapeutic drugs ineffective [37,80]. Comparison of human lung and colon cancer specimens with patient-matched normal tissues reveals a selective downregulation of rpL3 in the cancer tissues demonstrating that rpL3 is implicated in lung and colon cancer tumorigenesis [92,93]. In particular, the rpL3 mRNA amount decreases with malignant progression and the intensity of its expression is inversely related to tumor grade. These findings suggest that alterations in rpL3 expression promote tumor progression.

In addition, a correlation between rpL3 and the Bcl-2/Bax ratio in patients has been uncovered. Tumor proliferation, expressed as the ratio of *Bcl-2* mRNA copy number to that of *Bax*, is found to be inversely proportional to the decrease of *rpL3* gene expression and increased with tumor grade.

These results led us to propose a novel combined therapy with the use of 5-FU plus rpL3 in order to establish individualized therapy by examining rpL3 and p53 profiles in tumors to yield better clinical outcomes. Recently, our group has developed novel biodegradable nanoparticles (NP) as a platform to deliver the conventional drug 5-FU and the proapoptotic protein rpL3 aimed to enhance drug-induced cancer cell cytotoxicity [94]. Combined NP treatment results are more effective in inducing apoptosis in p53 null cancer cells than 5-FU or rpL3 alone, thereby providing a proof of principle on molecular-based individualized target therapy for the treatment of human colon cancer lacking p53 and rpL3.
